# Navigated intraoperative ultrasound in pediatric brain tumors

**DOI:** 10.1007/s00381-024-06492-8

**Published:** 2024-06-11

**Authors:** Kevin Klein Gunnewiek, Kirsten M. van Baarsen, Evie H. M. Graus, Wyger M. Brink, Maarten H. Lequin, Eelco W. Hoving

**Affiliations:** 1 Department of Neuro-Oncology, Princess Máxima Center for Pediatric Oncology, Utrecht, The Netherlands; 2 Magnetic Detection and Imaging Group, TechMed Centre, University of Twente, Enschede, The Netherlands

**Keywords:** Intraoperative ultrasound, Intraoperative MRI, Navigation, Pediatrics, Brain tumor

## Abstract

**Purpose:**

The aim of this study was to evaluate the diagnostic value and accuracy of navigated intraoperative ultrasound (iUS) in pediatric oncological neurosurgery as compared to intraoperative magnetic resonance imaging (iMRI).

**Methods:**

A total of 24 pediatric patients undergoing tumor debulking surgery with iUS, iMRI, and neuronavigation were included in this study. Prospective acquisition of iUS images was done at two time points during the surgical procedure: (1) before resection for tumor visualization and (2) after resection for residual tumor assessment. Dice similarity coefficients (DSC), Hausdorff distances 95th percentiles (HD95) and volume differences, sensitivity, and specificity were calculated for iUS segmentations as compared to iMRI.

**Results:**

A high correlation (*R* = 0.99) was found for volume estimation as measured on iUS and iMRI before resection. A good spatial accuracy was demonstrated with a median DSC of 0.72 (IQR 0.14) and a median HD95 percentile of 4.98 mm (IQR 2.22 mm). The assessment after resection demonstrated a sensitivity of 100% and a specificity of 84.6% for residual tumor detection with navigated iUS. A moderate accuracy was observed with a median DSC of 0.58 (IQR 0.27) and a median HD95 of 5.84 mm (IQR 4.04 mm) for residual tumor volumes.

**Conclusion:**

We found that iUS measurements of tumor volume before resection correlate well with those obtained from preoperative MRI. The accuracy of residual tumor detection was reliable as compared to iMRI, indicating the suitability of iUS for directing the surgeon’s attention to areas suspect for residual tumor. Therefore, iUS is considered as a valuable addition to the neurosurgical armamentarium.

**Trial registration number and date:**

PMCLAB2023.476, February 12th 2024.

## Introduction

Pediatric central nervous system tumors are known for having a poor prognosis and are the most common cause of death among all types of childhood cancers [1]. Surgical resection or debulking forms the first step in the treatment of most brain tumor types [2–4]. Typical surgical goals include cytoreduction, collection of tumor tissue for pathology assessment, or tumor mass debulking. An increase in the extent of resection (EoR) is associated with prolonged survival in both the adult and pediatric patient population [5–8]. Therefore, surgeons strive for the highest EoR without inducing major neurological deficits. Anatomical deformations and reliable identification of tumor-tissue boundaries during the procedure complicate this trade-off.

Intraoperative MRI (iMRI) and intraoperative ultrasound (iUS) are often used to evaluate the EoR during surgery. Both techniques enable intraoperative visualization of the tumor to assess whether the surgical goal is reached and to update the neuronavigation with new data. Performing iMRI is known to burden the surgical workflow as a time- and resource-intensive modality, both due to acquisition time as well as due to the required patient preparation and logistics involved. On the other hand, iUS does not provide a synoptic view of the brain and image quality is often inferior to MRI, hampering clinical interpretation. This is partially due to the more central location of pediatric tumors, which compromises the spatial resolution and image quality due to signal attenuation, as well as drainage of cerebrospinal fluid leading to inconsistencies in tissue-transducer contact and image artifacts.

The role of iUS has been described previously in pediatric neurosurgery literature, however without the use of neuronavigation and iMRI [9, 10]. The hypothesis is that integration of iUS with neuronavigation based on preoperative MRI can improve interpretability of iUS during surgery [11]. However, iUS remains prone to artifacts and iMRI is therefore still regarded as the gold standard in detecting residual tumor during surgery [12]. In literature, most studies focused on the value of either one of the two modalities. Only two recent studies report on the added value of integrating neuronavigation with iUS and iMRI [13, 14].

The aim of this study is therefore to evaluate the diagnostic value and accuracy of iUS in pediatric oncological neurosurgery with the ultimate goal to improve the EoR with a minimal burden on the surgical workflow. This study compares navigated iUS to preoperative MRI in terms of tumor visualization before resection and to iMRI in terms of residual tumor visualization.

## Methods

### Patient population and inclusion criteria

Pediatric patients undergoing an image guided neurosurgical tumor resection procedure involving iUS, iMRI, and neuronavigation between March 23 and December 28, 2023, at the Princess Máxima Center for Pediatric Oncology, Utrecht, the Netherlands, were eligible for inclusion. Exclusion criteria were (1) no neuronavigation, iUS, or iMRI available or (2) severe artifacts not allowing image interpretation. The study was approved by our Biobank and Data Access Committee under number PMCLAB2023.476.

### Data acquisition

In this study, iUS images are acquired at two time points during the surgical procedure. The two times points were based on the acquisition protocol proposed in a study by Bastos et al. [13]. When both iMRI and iUS were clinically indicated, the acquisition protocol was used. The first iUS acquisition was performed just before opening of the dura; this acquisition is called iUS1. Optional intermediate iUS acquisitions were performed upon request of the surgeon to monitor the resection progress, but these were not further evaluated in this study. The final iUS acquisition was performed just before iMRI scanning; this acquisition is called iUS2.

### Intraoperative ultrasound

**Fig. 1:**
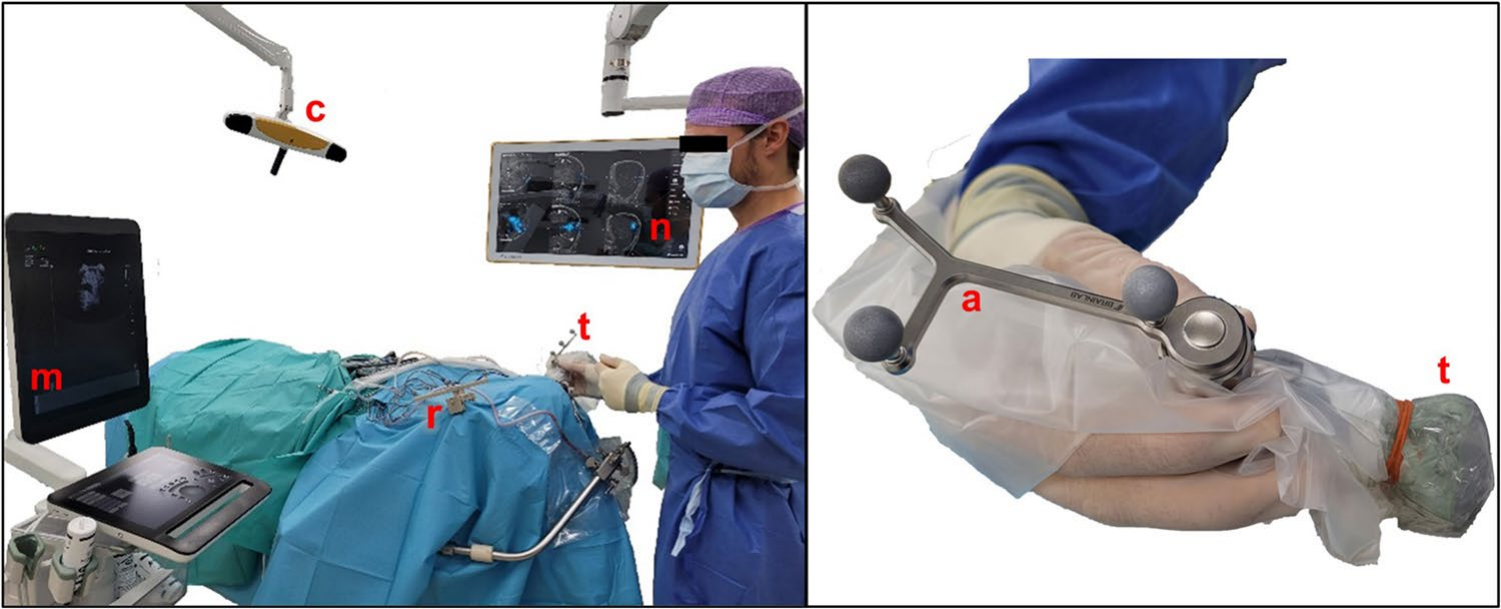
Navigated intraoperative acquisition set-up. Left image: set-up during iUS acquisition. Reference array (r) and transducer (t) are both optically tracked by stereotactic camera (c). The live ultrasound image is shown on the ultrasound machine (m) and the navigated image is overlaid on MRI (n). Right image: N13C5 transducer (BK5000, BK Medical, Denmark) (t), draped in a sterile cover with the sterile array (a) for optical navigation is attached (Brainlab, Munich, Germany)

The intraoperative ultrasound acquisitions were performed according to a predefined standardized protocol with a 2D neuro-cranial curvilinear transducer (N13C5, BK5000, BK Medical, Denmark), which has a frequency range of 13–5 MHz and a surface area of 29 × 10 mm. The initial parameters for iUS acquisition were set to a frequency of 10 MHz and a sector width of 140%. Besides, auto-gain was enabled. An initial estimation of the acquisition depth was based on a depth measurement in the pre-surgical planning software (Brainlab, Munich, Germany), which typically resulted in an acquisition depth between 7 and 11 cm. The sterile reference array is mounted to the ultrasound transducer and is tracked by the Brainlab Buzz Navigation system (Brainlab, Munich, Germany). Before creation of a 3D volume, an initial exploration of the tumor in “live mode” was done. This allowed for fine-tuning of the initial acquisition parameters. The acquisition frequency was adjusted when the contrast of deeper structures was not satisfactory, by decreasing the frequency to 8 MHz. The set-up for iUS acquisition is shown in Fig. 1.

Integration of iUS with neuronavigation allows for 3D volume reconstruction of consecutive 2D B-mode iUS images in the navigated iUS module (Brainlab, Munich, Germany). For neuronavigation initialization, patient-to-image registration was either based on iMRI or surface matching depending on the clinical indication. The resolution of the 2D images was 0.24 × 0.24 mm in-plane. The 3D volume was automatically reconstructed from consecutive 2D images with a slice thickness of 1 mm. Depending on the number of acquired 2D images, volume reconstruction took up to 1 min. The 3D volume reconstruction captured the entire tumor volume. After the first iUS acquisition, the initial scanning parameters were kept identical for additional scans. Only the frequency was decreased, if necessary. During each iUS acquisition, at least two volumes were acquired, each in a different orthogonal imaging plane.

### Intraoperative MRI

Two different MRI acquisitions were defined for comparison with the iUS volumes: one before and one during tumor resection. Each patient was scanned at least 1 day before surgery. Although interhospital differences were present in preoperative imaging protocols, the protocols always contained T1-weighted imaging with and without gadolinium administration as well as T2-weighted imaging. In the intraoperative setting, patients were scanned with a 3 T 70 cm bore MRI scanner (Ingenia Elition X, Philips Healthcare, Best, the Netherlands). The intraoperative T1-weighted contrast enhanced images were acquired with a 3D MPRAGE sequence using a TI/TR/TE = 681/6.5/3.0 ms, a flip angle of 6°, and acquired isotropic resolution of 1.25 mm. The intraoperative T2-weighted images were acquired with a 3D TSE sequence using a TR/TE = 3000/280 ms, a refocusing control angle of 40°, and acquired isotropic resolution of 1 mm.

### Clinical assessment of residual tumor

After acquisition of the iUS2 volume (just before the iMRI acquisition), the surgeon assessed the iUS image for the presence of residual tumor. If the iUS images were inconclusive, a neuroradiologist was consulted for assessment. The presence of residual tumor on iMRI was then evaluated by the surgeon in consultation with the neuroradiologist. Based on this interpretation, the surgeon decided whether continuation of tumor resection was necessary and safe.

### Creating segmentations and quantitative analysis

**Fig. 2:**
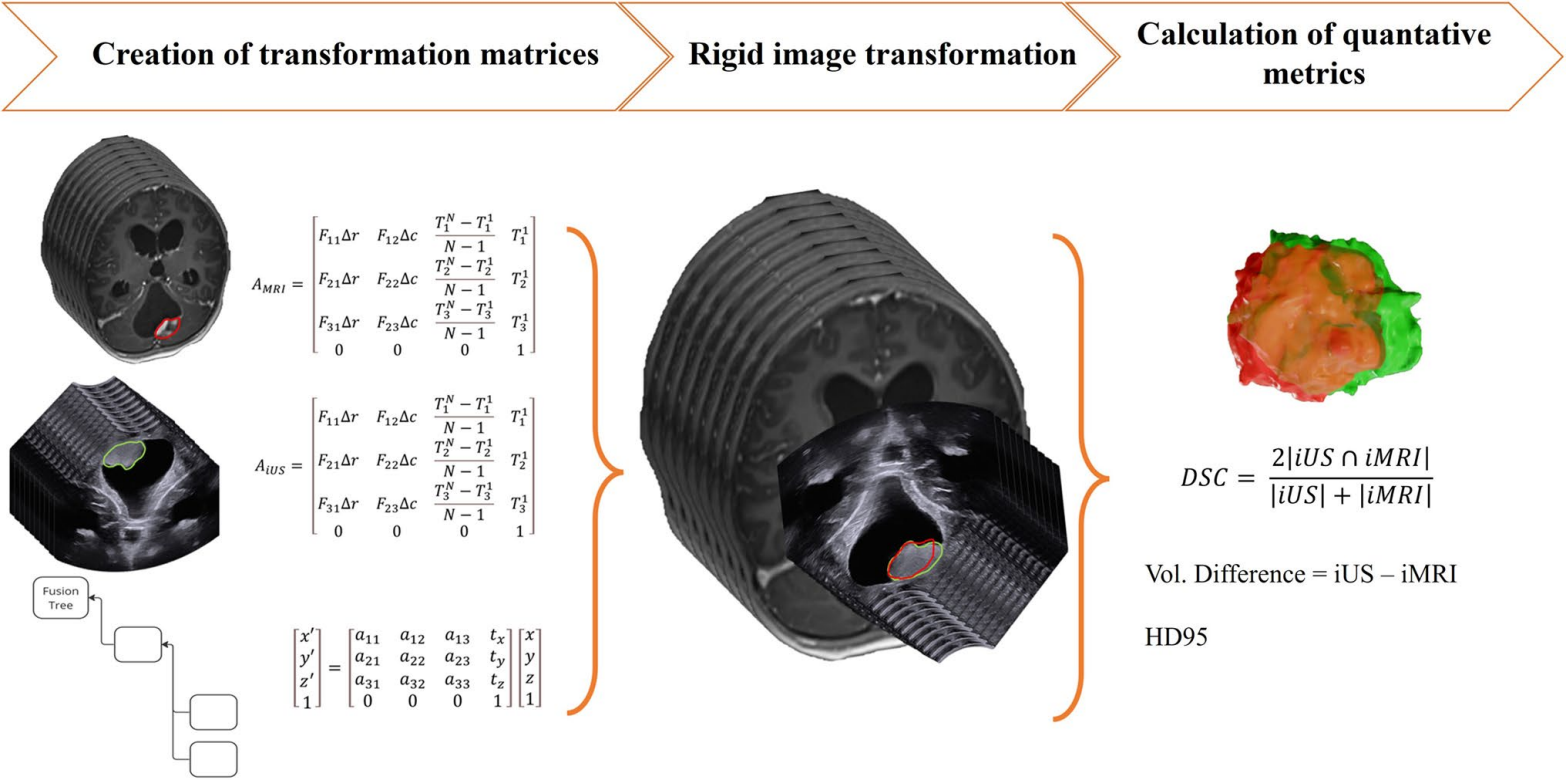
Workflow of data processing and analysis is divided into (1) creation of transformation matrices, multiplying matrices to obtain one transformation to the destination image; (2) rigid image transformation, applying the transformation matrix; and (3) calculation of quantitative metrics, i.e., volume difference, Dice similarity coefficient (DSC), and Hausdorff distance 95th percentile (HD95)

A quantitative data-analysis was performed on both the iUS1 and iUS2 volumes to evaluate the structural and positional similarity. An overview of the analysis pipeline is shown in Fig. 2.

First, out of the two iUS acquisitions, the volume with highest image quality was selected per patient. When artifacts or incomplete acquisitions impeded tumor visualization in both orthogonal images, the acquisition was excluded from further analysis. Segmentation of solid tumor components was segmented with the use of a semi-automatic segmentation tool (Smart Brush, Brainlab, Munich, Germany). Large solitary cystic components (> 2 cm^3^) were excluded from the segmentation.

MRI segmentations are based on T1-weighted gadolinium enhanced images, which are clinically used to plan (additional) tumor resection. To verify the segmentation of non-enhancing tumor regions, T2-weighted imaging was used as a control. The tumor volumes were segmented in the iUS volumes first, to minimize information bias. Images and segmentations from both modalities were exported to DICOM (Digital Imaging and Communications in Medicine) image format for further analysis.

Data analysis was executed using a custom processing pipeline implemented in Python (version 3.7) for quantitative analysis of the segmentations. Transformation matrices were retrieved from the exported DICOM data and applied to the source images and corresponding segmentations for quantitative comparison between iUS and MRI.

The Dice similarity coefficient (DSC), Hausdorff distance (HD), and absolute volume difference were evaluated to quantify the spatial accuracy of iUS in detecting and characterizing tumor tissue. The DSC provides an indication of the degree of overlap, combining volumetric and positional information (see Eq. 1). The degree of overlap is inherent to the quality of the segmentation, since the volume and morphology are affected. Besides, the quality of the registration for neuronavigation is important for optimal positioning of the navigated iUS on the MRI, therefore also affecting the DSC. A DSC ranging between 0.7 and 0.9 is considered as a good similarity of the volumes between the both modalities.

1 $$DSC=\frac{2|iUS\cap iMRI|}{|iUS|+|iMRI|}$$

As the DSC is sensitive for small volumes, Hausdorff distances and volume errors were also evaluated. The Hausdorff distance describes the Euclidean distance between every individual point in one volume to the closest point in the other volume. The maximum Hausdorff distance is prone to outliers; therefore, the 95th percentile is calculated (HD95). An HD95 smaller than 10 mm was considered as clinically relevant, approaching the surgical margins as described in literature and by surgical experience [15, 16]. The absolute volume difference is defined as the absolute difference between the iUS and iMRI volume.

Based on previous studies [9, 10, 13, 14, 17–22], we defined that iUS has a good diagnostic value in detecting residual tumor if a sensitivity and specificity of more than 80% was achieved.

## Results

Between March 23 and December 28, 2023, 24 patients were included. The mean age was 7.6 years (± SD 3.9), with the most occurring tumor type being pilocytic astrocytoma (*n* = 9). Further patient characteristics are provided in Table 1.

**Table 1:** Patient population characteristics

#	Sex	Age (years)	Tumor type	Tumor location	Residual on iMRI	Surgical position	Tumor volume (preop-MRI) [ml]
1	Male	12	Pilocytic astrocytoma	Hemispheric	n	Supine	59.4
2	Female	4	Ependymoma	Hemispheric	n	Supine	63.3
3	Male	8	Low grade glioma	Hemispheric	n	Supine	24.4
4	Female	6	Pilocytic astrocytoma	Hemispheric	y	Supine	32.4
5	Male	18	Low grade glioma	Hemispheric	n	Supine	0.884
6	Male	10	DNET	Hemispheric	y	Supine	7.76
7	Female	2	High grade glioma	Hemispheric	y	Supine	77.0
8	Male	5	DNET	Hemispheric	n	Supine	18.3
9	Female	3	Ganglioglioma	Infratentorial	y	Prone	38.6
10	Female	9	Medulloblastoma	Infratentorial	y	Prone	21.3
11	Male	10	Pilocytic astrocytoma	Infratentorial	y	Prone	6.17
12	Female	3	Medulloblastoma	Infratentorial	y	Prone	21.1
13	Male	9	Pilocytic astrocytoma	Infratentorial	n	Prone	3.68
14	Male	2	Ependymoma	Infratentorial	n	Prone	107
15	Male	13	Medulloblastoma	Infratentorial	n	Prone	0.347
16	Female	3	Pilocytic astrocytoma	Infratentorial	y	Prone	42.9
17	Male	7	Pilocytic astrocytoma	Infratentorial	n	Prone	25.3
18	Male	4	Pilocytic astrocytoma	Infratentorial	n	Prone	17.7
19	Male	9	Pilocytic astrocytoma	Intraventricular	y	Supine	3.30
20	Male	13	Giant cell astrocytoma	Intraventricular	n	Supine	1.10
21	Male	9	Pilocytic astrocytoma	Suprasellar	y	Supine	20.3
22	Female	7	Craniopharyngioma	Suprasellar	n	Supine	37.4
23	Male	9	Craniopharyngioma	Suprasellar	n	Supine	0.531
24	Male	7	Craniopharyngioma	Suprasellar	n	Supine	16.7

*DNET* dysembryoplastic neuroepithelial tumor

**Fig. 3:**
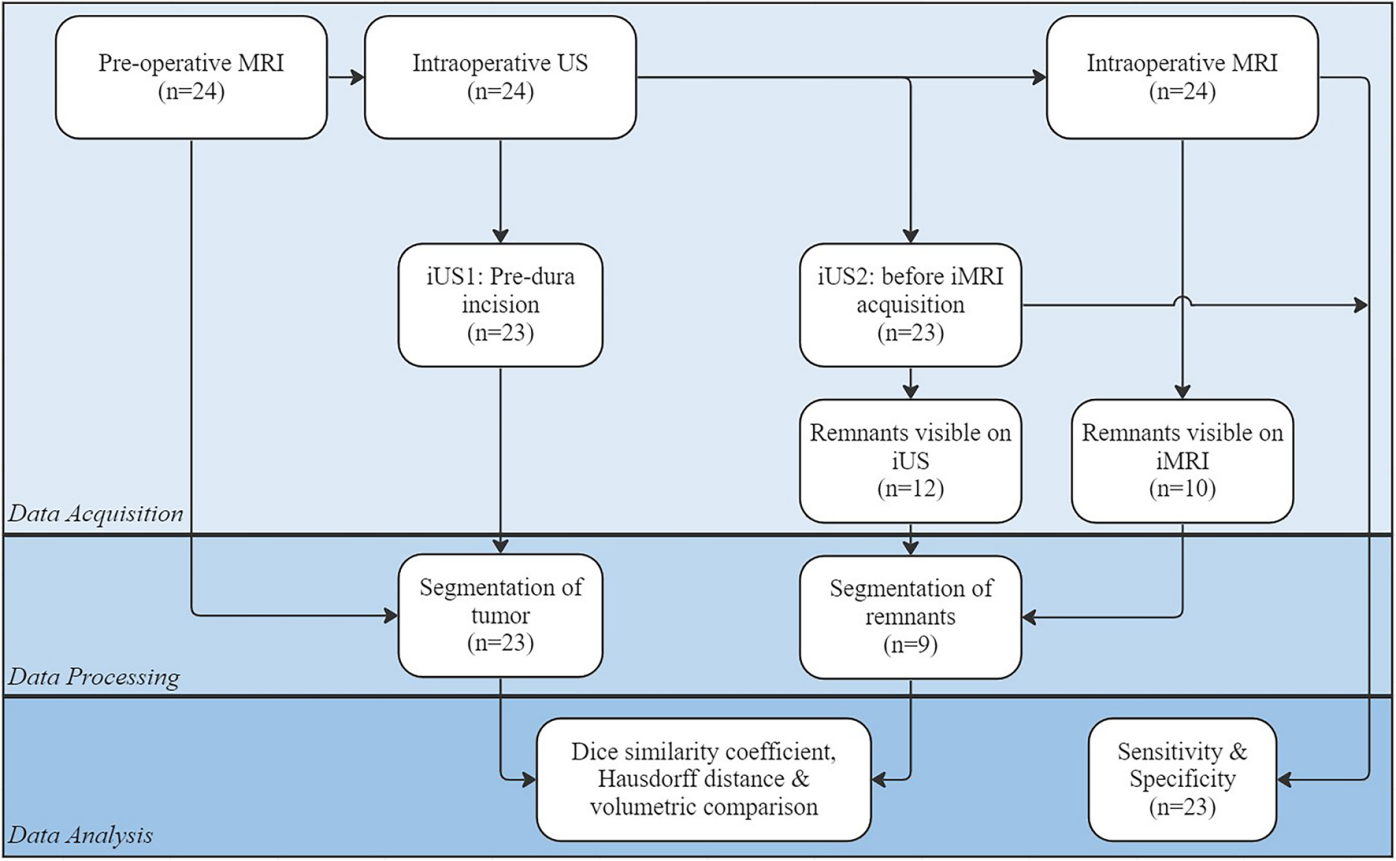
Flowchart showing different acquisition, processing, and analysis steps with the number of patients involved

The inclusion flowchart is shown in Fig. 3. Two patients were excluded from intraoperative image analysis due to image artifacts and unavailability of iUS. Fourteen patients showed no residual tumor on iMRI and were therefore not included for further quantitative analysis. A total of nine patients were included for quantitative analysis of intraoperative imaging.

Median tumor volumes were 20.3 cm^3^ (IQR 29.9 cm^3^) as measured on T1-weighted contrast enhanced MRI. The iMRI-based patient-to-image registration was the most used method (*n* = 13).

Residual tumor was detected on iUS2 images in 12 patients. In 10 of these cases, residual tumor was also seen on iMRI, which yielded a sensitivity of 100% and a specificity of 84.6%. From these 10 cases, one case was excluded from further quantitative analysis due to poor image quality. A mean extent of resection (EoR) of 74.7% was determined for the other 9 cases.

### Tumor characterization: iUS1

**Fig. 4:**
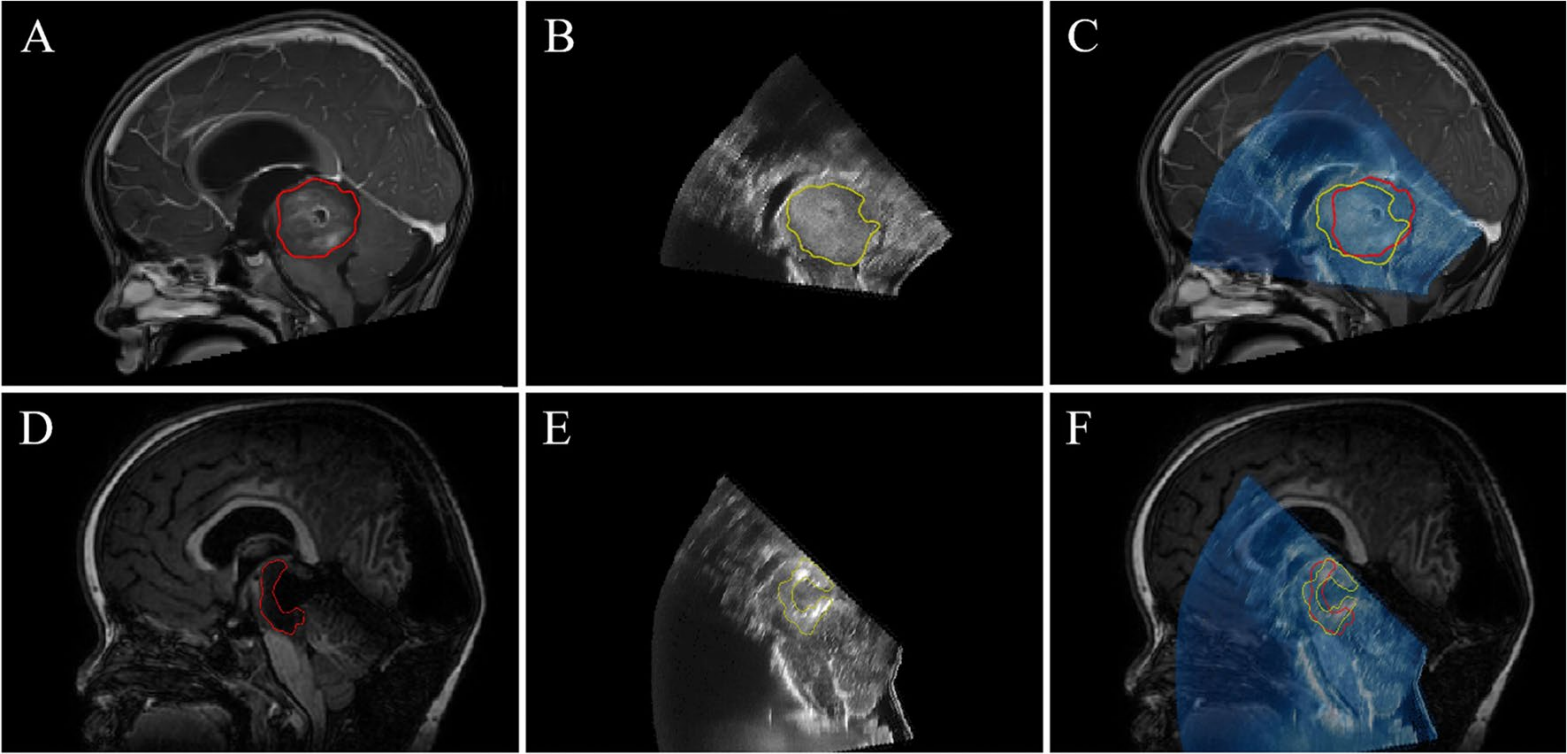
Example segmentations in preoperative and intraoperative MRI and US images. The first row shows the tumor segmentation in **A** preoperative MRI, **B** iUS1, and **C** for both modalities in an overlay. The second row shows the remnant segmentation in **D** intraoperative MRI, **E** iUS2, and **F** for both modalities in an overlay

**Fig. 5:**
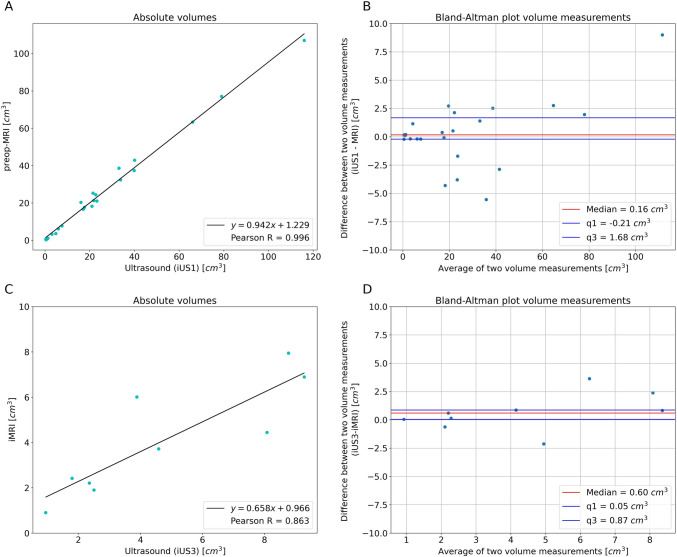
Volume based metrics of iUS acquisitions compared to MRI. **A** The absolute volume plotted per patient for iUS1 and preoperative MRI. A regression line is plotted and shows a coefficient of 0.942. The Pearson correlation coefficient is 0.996. **C** The absolute volume plotted per patient with a remnant visible on both iUS2 and iMRI. The regression line coefficient of 0.658 and a Pearson correlation coefficient of 0.863 were found. **B** and **D** Bland–Altman plots, in which the differences between iUS and MRI are plotted against their averages. **B** The tumor volumes before resection and **D** remnant volumes as seen on iUS2 and iMRI. Median volume differences of 0.16 cm^3^ and 0.60 cm^3^ were found for iUS1 and iUS2, respectively. The first and third quartiles are shown in blue

Tumor segmentations were created in both the iUS1 and preoperative T1-weighted contrast enhanced MRI data in 23 patients. Example segmentations are shown in Fig. 4A–C. Quantitative analysis of the spatial correspondence yielded a median DSC of 0.72 (IQR 0.14) and a median HD95 of 4.98 mm (IQR 2.22 mm). The measured volumes ranged from 0.35 to 107.0 cm^3^. A high correlation was found between the tumor volume measurements of iUS1 segmentations when compared to those derived from preoperative MRI (*R* = 0.99), as shown in Fig. 5A. A median volume difference of 0.16 cm^3^ (IQR 1.89 cm^3^) was found, which shows neither a trend towards over- or underestimation of the volume on iUS1 as compared to the preoperative MRI. The median absolute volume difference was 1.40 cm^3^ (IQR 2.54 cm^3^), as shown in the Bland–Altman plot in Fig. 5B. Similar analyses were performed using preoperative T2-weighted MRI images, yielding comparable results.

### Residual tumor detection: iUS2

Tumor segmentations created for 9 patients in both iUS2 and intraoperative T1-weighted contrast enhanced MRI yielded a median DSC of 0.58 (IQR 0.27) and a median HD95 of 5.84 mm (IQR 4.04 mm) were determined. Example segmentations are shown in Fig. 4D–F. The residual tumor volumes ranged from 0.90 to 7.95 cm^3^. Overall, a slight overestimation of the residual tumor volume was observed on iUS as compared to iMRI, as shown in Fig. 5C. A lower Pearson correlation coefficient of 0.863 was found as compared to the iUS1 findings. A median absolute volume difference of 0.60 cm^3^ (IQR 0.92 cm^3^) was found, as shown in Fig. 5D. The median absolute volume difference was 0.82 cm^3^ (IQR 1.52 cm^3^). Similar analyses performed using to T2-weighted iMRI images, yielding comparable results.

## Discussion

Our study demonstrated that iUS has good diagnostic performance in detecting residual tumor and has a high spatial accuracy of visualizing (residual) tumor tissue when compared to iMRI. Regarding the first iUS acquisition (iUS1), the calculated tumor volumes showed an excellent correlation and a good spatial correspondence was observed when compared to preoperative MRI. Regarding the second iUS acquisition (iUS2), the relative volume difference (25.7%) was higher when compared to iUS1 (7.01%). In our current results, lower DSC values were found for the iUS2 volumes when compared to iUS1. This was to be expected as this metric is more sensitive when evaluating smaller volumes. Nevertheless, the performance of iUS in localization of residual tumor is considered promising.

Multiple studies have reported that navigated iUS shows to be a good addition to the neurosurgical armamentarium [9, 13, 14, 17–21]. There is not yet a clear consensus in literature on the sensitivity and specificity of iUS in detecting residual tumor. For the adult population, sensitivity and specificity values found in other studies [11, 14, 23] vary between 46 and 94% and 83 and 100%, respectively. Only one study by Carai et al. [9] describes these metrics for a large pediatric population (*n* = 154) with a sensitivity and specificity of 86% and 99%. Our study population is significantly smaller, which made that a small number of inconclusive cases substantially affected the diagnostic metrics. For example, two inconclusive cases were considered positive for residual tumor, leading to a specificity lower than reported by other studies. However, false positives for residual tumor will not directly lead to resection, but rather directs the surgeon’s attention to the suspected area.

Few studies have reported quantitatively on the spatial accuracy of iUS. Most studies report merely on good visual congruency between images and the added diagnostic value of iUS in detecting residual tumor. These findings were confirmed in our research by the high correlation between tumor volumes on iUS and iMRI and the good DSC and HD95.

This study demonstrated that navigated iUS showed to be a very promising addition to the neurosurgical armamentarium in detecting tumor tissue before and during resection. Without major disruptions of the surgical workflow, iUS can intermittently provide the surgeon with additional information for interactive guidance to localize potential residual tumor. Besides, iUS acquisitions are safer, briefer, and require less patient preparation than those for iMRI. Image reconstruction based on navigated iUS allowed surgeons to interpret images better as compared to unnavigated 2D imaging. The integration with neuronavigation makes iUS usage more effective. Based on our clinical experiences, iUS could lead to more efficient timing of iMRI acquisitions or be a substitute when iMRI is not available.

Limitations in this study were the low number of inclusions (*n* = 24) and the low number of surgeons acquiring and assessing the images (*n* = 2). Another limitation was a selection bias inevitably introduced by allocating the iMRI mostly to patients with complex procedures, due to logistic constraints. Additionally, the image quality of iUS2 was affected by surgery, resulting in generally poorer images than those acquired before tumor debulking. As described by multiple studies [12, 19, 24, 25], acoustic enhancing artifacts (AEA), contusion, and edema are hyperechoic and could be mistaken for residual tumor, which could have led to suboptimal tumor segmentations in the iUS2 images. Clinically, this does not necessarily lead to additional tumor resection, but rather a direction of the surgeon’s attention to the suspected area. For an optimal comparison of intraoperative images, we would like to stress that iUS image acquisition before dura opening (iUS1) is required. Furthermore, to optimize image quality, it is advised to (1) acquire images transcortically, (2) maintain proper transducer-tissue contact, and (3) to position the patient in such a way as to avoid fluid drainage from the resection cavity. Lastly, this study was limited to the assessment and quantification of the diagnostic value of conventional iUS imaging based on B-mode images. Several recent studies report on the potential of more advanced iUS techniques like Doppler imaging [18], strain elastosonography [26, 27], and contrast-enhanced ultrasound (CEUS) [19, 28–32]. Future research needs to show if more advanced iUS techniques contribute to a more optimal EoR and a minimal disruption of the surgical workflow.

## Conclusions

This study demonstrated that iUS performs well in visualizing brain tumors during pediatric neurosurgery before and after debulking. A good similarity was found between iUS and MRI. iUS can direct the surgeon’s attention to areas suspect for residual tumor in a large resection cavity. Based on our findings, iUS is considered a promising addition to the neurosurgical armamentarium.

## Data Availability

The data that support the findings of this study are not openly available due to reasons of patient confidentiality and are available from the corresponding author upon reasonable request. Data are located in controlled access data storage at Princess Máxima Center.

## References

[1] Udaka YT, Packer RJ (2018) Pediatric brain tumors. Neurol Clin 36(3):533–556. 10.1016/J.NCL.2018.04.009 30072070 10.1016/j.ncl.2018.04.009

[2] Rudà R, Reifenberger G, Frappaz D et al (2018) EANO guidelines for the diagnosis and treatment of ependymal tumors. Neuro Oncol 20(4):445–456. 10.1093/NEUONC/NOX166 29194500 10.1093/neuonc/nox166 PMC5909649

[3] Gnekow AK, Kandels D, Van TC et al (2019) SIOP-E-BTG and GPOH guidelines for diagnosis and treatment of children and adolescents with low grade glioma. Klin Padiatr 231(3):107–135. 10.1055/A-0889-8256 31108561 10.1055/a-0889-8256

[4] Srinivasan V, Ghali M, North R, Boghani Z, Hansen D, Lam S (2016) Modern management of medulloblastoma: molecular classification, outcomes, and the role of surgery. Surg Neurol Int 7(Suppl 44):S1135–S1135. 10.4103/2152-7806.196922 28194300 10.4103/2152-7806.196922 PMC5299153

[5] Zipfel J, Tellermann J, Besch D et al (2022) Surgical management of pre-chiasmatic intraorbital optic nerve gliomas in children after loss of visual function-resection from bulbus to chiasm. Children (Basel) 9(4). 10.3390/CHILDREN9040459 10.3390/children9040459 PMC9029433 35455503

[6] Revilla-Pacheco F, Rodríguez-Salgado P, Barrera-Ramírez M et al (2021) Extent of resection and survival in patients with glioblastoma multiforme: systematic review and meta-analysis. Medicine 100(25):e26432–e26432. 10.1097/MD.0000000000026432 34160432 10.1097/MD.0000000000026432 PMC8238332

[7] Sanai N, Polley MY, McDermott MW, Parsa AT, Berger MS (2011) An extent of resection threshold for newly diagnosed glioblastomas: clinical article. J Neurosurg 115(1):3–8. 10.3171/2011.2.JNS10998 21417701 10.3171/2011.2.jns10998

[8] Fujii Y, Muragaki Y, Maruyama T et al (2018) Threshold of the extent of resection for WHO grade III gliomas: retrospective volumetric analysis of 122 cases using intraoperative MRI. J Neurosurg 129(1):1–9. 10.3171/2017.3.JNS162383 28885120 10.3171/2017.3.JNS162383

[9] Carai A, De Benedictis A, Calloni T et al (2021) Intraoperative ultrasound-assisted extent of resection assessment in pediatric neurosurgical oncology. Front Oncol 11. 10.3389/FONC.2021.660805 10.3389/fonc.2021.660805 PMC8097032 33968768

[10] Moiyadi AV, Shetty P, Degaonkar A (2017) Resection of pediatric brain tumors: intraoperative ultrasound revisited. J Pediatr Neurosci 12(1):19. 10.4103/JPN.JPN_141_16 28553373 10.4103/jpn.JPN_141_16 PMC5437781

[11] de Quintana-Schmidt C, Salgado-Lopez L, Aibar-Duran JA et al (2022) Neuronavigated ultrasound in neuro-oncology: a true real-time intraoperative image. World Neurosurg 157:e316–e326. 10.1016/J.WNEU.2021.10.082 34655818 10.1016/j.wneu.2021.10.082

[12] Šteňo A, Buvala J, Babková V, Kiss A, Toma D, Lysak A (2021) Current limitations of intraoperative ultrasound in brain tumor surgery. Front Oncol 11:851. 10.3389/FONC.2021.659048/BIBTEX 10.3389/fonc.2021.659048 PMC8019922 33828994

[13] Bastos DCDA, Juvekar P, Tie Y et al (2021) Challenges and opportunities of intraoperative 3D ultrasound with neuronavigation in relation to intraoperative MRI. Front Oncol 11:1463. 10.3389/FONC.2021.656519/BIBTEX 10.3389/fonc.2021.656519 PMC8139191 34026631

[14] Hou Y, Tang J (2022) Advantages of using 3D intraoperative ultrasound and intraoperative MRI in glioma surgery. Front Oncol 12:2743. 10.3389/FONC.2022.925371/BIBTEX 10.3389/fonc.2022.925371 PMC9203997 35719958

[15] Young JS, Morshed RA, Hervey-Jumper SL, Berger MS (2023) The surgical management of diffuse gliomas: current state of neurosurgical management and future directions. Neuro Oncol. Published online July 2023. Erratum in: Neuro Oncol 26(6):1175: 10.1093/neuronc/noae083, 10.1093/neuonc/noad133 10.1093/neuonc/noad133 PMC10708937 37499054

[16] Moiraghi A, Prada F, Delaidelli A et al (2020) Navigated intraoperative 2-dimensional ultrasound in high-grade glioma surgery: impact on extent of resection and patient outcome. Oper Neurosurg (Hagerstown) 18(4):363–373. 10.1093/ONS/OPZ203 31435672 10.1093/ons/opz203

[17] Yeole U, Singh V, Mishra A, Shaikh S, Shetty P, Moiyadi A (2020) Navigated intraoperative ultrasonography for brain tumors: a pictorial essay on the technique, its utility, and its benefits in neuro-oncology. Ultrasonography 39(4):394. 10.14366/USG.20044 32660206 10.14366/usg.20044 PMC7515658

[18] Saß B, Pojskic M, Zivkovic D, Carl B, Nimsky C, Bopp MHA (2021) Utilizing intraoperative navigated 3D color Doppler ultrasound in glioma surgery. Front Oncol 11:3122. 10.3389/FONC.2021.656020/BIBTEX 10.3389/fonc.2021.656020 PMC8416533 34490080

[19] Dixon L, Lim A, Grech-Sollars M, Nandi D, Camp S (2022) Intraoperative ultrasound in brain tumor surgery: a review and implementation guide. Neurosurg Rev 45(4):2503–2515. 10.1007/S10143-022-01778-4/FIGURES/6 35353266 10.1007/s10143-022-01778-4 PMC9349149

[20] Hou Y, Li Y, Li Q, Yu Y, Tang J (2022) Full-course resection control strategy in glioma surgery using both intraoperative ultrasound and intraoperative MRI. Front Oncol 12:4503. 10.3389/FONC.2022.955807/BIBTEX 10.3389/fonc.2022.955807 PMC9453394 36091111

[21] Wirtz CR, Albert FK, Schwaderer M et al (2016) The benefit of neuronavigation for neurosurgery analyzed by its impact on glioblastoma surgery. Neurol Res 22(4):354-360.10.1080/01616412.2000.11740684 10.1080/01616412.2000.11740684 10874684

[22] Gerard IJ, Kersten-Oertel M, Hall JA, Sirhan D, Collins DL (2020) Brain shift in neuronavigation of brain tumors: an updated review of intra-operative ultrasound applications. Front Oncol 10:1. 10.3389/FONC.2020.618837 33628733 10.3389/fonc.2020.618837 PMC7897668

[23] Shetty P, Yeole U, Singh V, Moiyadi A (2021) Navigated ultrasound-based image guidance during resection of gliomas: practical utility in intraoperative decision-making and outcomes. Neurosurg Focus 50(1):1–10. 10.3171/2020.10.FOCUS20550 10.3171/2020.10.FOCUS20550 33386014

[24] Šteňo A, Buvala J, Šteňo J (2021) Large residual pilocytic astrocytoma after failed ultrasound-guided resection: intraoperative ultrasound limitations require special attention. World Neurosurg 150:140–143. 10.1016/J.WNEU.2021.03.138 33819702 10.1016/j.wneu.2021.03.138

[25] Culleton S, McKenna B, Dixon L et al (2021) Imaging pitfalls in paediatric posterior fossa neoplastic and non-neoplastic lesions. Clin Radiol 76(5):391.e19-391.e31. 10.1016/J.CRAD.2020.12.011 33648757 10.1016/j.crad.2020.12.011

[26] Cepeda S, García-García S, Arrese I et al (2021) Comparison of intraoperative ultrasound B-mode and strain elastography for the differentiation of glioblastomas from solitary brain metastases. An automated deep learning approach for image analysis. Front Oncol 10:3322. 10.3389/FONC.2020.590756/BIBTEX 10.3389/fonc.2020.590756 PMC7884775 33604286

[27] Prada F, Ciocca R, Corradino N et al (2022) Multiparametric intraoperative ultrasound in oncological neurosurgery: a pictorial essay. Front Neurosci 16:881661. 10.3389/FNINS.2022.881661/BIBTEX 35516800 10.3389/fnins.2022.881661 PMC9063404

[28] Hwang M, Barnewolt CE, Jüngert J, Prada F, Sridharan A, Didier RA (2021) Contrast-enhanced ultrasound of the pediatric brain. Pediatr Radiol 51(12):2270–2283. 10.1007/S00247-021-04974-4 33599780 10.1007/s00247-021-04974-4 PMC11458139

[29] Prada F, Del Bene M, Fornaro R et al (2016) Identification of residual tumor with intraoperative contrast-enhanced ultrasound during glioblastoma resection. Neurosurg Focus 40(3):E7–E7. 10.3171/2015.11.FOCUS15573 26926065 10.3171/2015.11.FOCUS15573

[30] Squires JH, McCarville Beth M (2021) Contrast-enhanced ultrasound in children: implementation and key diagnostic applications. Am J Roentgenol 217(5):1217–1230. 10.2214/AJR.21.25713 33908269 10.2214/AJR.21.25713

[31] Pepa Della GM, Ius T, La Rocca G et al (2020) 5-Aminolevulinic acid and contrast-enhanced ultrasound: the combination of the two techniques to optimize the extent of resection in glioblastoma surgery. Neurosurgery. 86(6):E529–E540. 10.1093/NEUROS/NYAA037 32186345 10.1093/neuros/nyaa037

[32] Della Pepa GM, Menna G, Ius T et al (2020) Contrast enhanced ultrasound (CEUS) applications in neurosurgical and neurological settings – new scenarios for brain and spinal cord ultrasonography. A systematic review. Clin Neurol Neurosurg 198:106105. 10.1016/J.CLINEURO.2020.106105 32739680 10.1016/j.clineuro.2020.106105

