# Correction: Navigated intraoperative ultrasound in pediatric brain tumors

**DOI:** 10.1007/s00381-025-06824-2

**Published:** 2025-04-14

**Authors:** Kevin Klein Gunnewiek, Kirsten M. van Baarsen, Evie H. M. Graus, Wyger M. Brink, Maarten H. Lequin, Eelco W. Hoving

**Affiliations:** 1 Department of Neuro-Oncology, Princess Máxima Center for Pediatric Oncology, Utrecht, The Netherlands; 2 Magnetic Detection and Imaging Group, Techmed Centre, University of Twente, Enschede, The Netherlands

**Correction: Child's Nervous System (2024) 40:2697–2705**

10.1007/s00381-024-06492-8

In this article, three sentences require correction.

1. “Thirteen patients showed no residual tumor on iMRI and were therefore not included for further quantitative analysis.” should be “Fourteen patients showed no residual tumor on iMRI and were therefore not included for further quantitative analysis.”
2. “A total of seven patients were included for quantitative analysis of intraoperative imaging."should be “A total of nine patients were included for quantitative analysis of intraoperative imaging.”
3. "Tumor segmentations were created in both the iUS1 and preoperative T1-weighted contrast enhanced MRI data in 24 patients."should be “Tumor segmentations were created in both the iUS1 and preoperative T1-weighted contrast enhanced MRI data in 23 patients.”

The original article has been corrected.

